# Using quantitative proteomic analysis to understand genotype specific intrinsic drug resistance in melanoma

**DOI:** 10.18632/oncotarget.263

**Published:** 2011-04-15

**Authors:** John M. Koomen, Keiran S. M. Smalley

**Affiliations:** ^1^ Program in Molecular Oncology, The Moffitt Cancer Center & Research Institute, Tampa, FL, USA; ^2^ Program in Experimental Therapeutics, The Moffitt Cancer Center & Research Institute, Tampa, FL, USA; ^3^ Program in Cutaneous Oncology, The Moffitt Cancer Center & Research Institute, Tampa, FL, USA; ^4^ The Comprehensive Melanoma Research Center, The Moffitt Cancer Center & Research Institute, Tampa, FL, USA

**Keywords:** Oncogene, cancer, kinase, BRAF V600E, drug resistance, proteomics

## Abstract

The discovery of activating *BRAF* V600E mutations in 50% of all melanoma patients and the development of small molecule BRAF inhibitors looks set to revolutionize the therapy of disseminated melanoma. However, in the recent clinical trial of the BRAF inhibitor, vemurafenib (PLX4032), a significant percentage of *BRAF* V600E mutant melanoma patients did not meet the RECIST criteria for a response. Recent work from our lab identified loss of the tumor suppressor phosphatase and tensin homolog (PTEN) as being a possible mediator of intrinsic BRAF inhibitor resistance. In this commentary, we describe the development of a novel mass spectrometry based proteomic screen of Bcl-2 family proteins that was used to delineate the PTEN-dependent differences in apoptosis signaling observed when BRAF was inhibited. We further discuss how use of these sensitive quantitative proteomic methods gives unique insights into the signaling of cancer cells that are not captured through routine biochemical techniques and how this may lead to the development of combination therapy strategies for overcoming intrinsic BRAF inhibitor resistance.

## TARGETED THERAPY IN MELANOMA

Melanoma is the deadliest form of skin cancer. It arises from the malignant transformation of melanocytes and has long been notorious for its resistance to chemotherapy, radiotherapy and immunotherapy. In recent years, great strides have been made in our understanding of the underlying genetic and biological basis of melanoma initiation and development. We now stand at an exciting juncture in melanoma research in which our accumulated knowledge about melanoma biology is translating into new therapeutic strategies. One key discovery of the last decade is the identification of activating mutations in the serine/threonine kinase BRAF in up to 50% of all melanomas [1]. There is now good evidence that mutated *BRAF* is a key initiating event in melanoma development and that continuous BRAF signaling is required for melanoma progression [2, 3]. Most of the transforming activity of mutant *BRAF* is mediated through the activation of the RAF/MEK/ERK signaling pathway which drives cell cycle dysregulation and uncontrolled growth by reducing expression of the cyclin dependent kinase inhibitor p27 and by increasing the expression of cyclin D1 [4, 5]. In addition to its effects upon cell growth, mutant *BRAF* also contributes to the oncogenic phenotype of melanoma cells through both down regulation of apoptotic signals and enhancement of cell invasion [6-9]. Recent clinical studies have demonstrated that the presence of a *BRAF* mutation is prognostic for melanoma and is associated with reduced survival in the metastatic setting [10].

The discovery of activating *BRAF* mutations in melanoma prompted a flurry of drug discovery activity and the development of small molecule BRAF inhibitors. The list of BRAF inhibitors currently undergoing preclinical and clinical evaluation includes XL281, SB590885, GDC-0879, GSK2118438, AZ628 and PLX4032 [11-14]. Of these, PLX4032 (vemurafenib) and its analog, PLX4720, have been most extensively studied [13, 15-18]. Treatment of melanoma cell lines and mouse xenografts with PLX4032/4720 led to both G1 phase cell cycle arrest and the induction of apoptosis [13, 15]. The effects of PLX4032 were noted to be *BRAF* mutation specific, and equivalent responses were seen in melanoma models with both heterozygous and homozygous *BRAF* mutations [13]. No anti-proliferative or cytotoxic effects were observed in melanoma cell cultures that lacked the *BRAF* mutation. Interestingly, not all *BRAF* mutated melanoma cell lines were similarly sensitive to PLX4032 and PLX4720 though, with some cell lines exhibiting intrinsic resistance [17-19].

In the phase I clinical trial, vemurafenib led to significant levels of tumor shrinkage in 80% of patients whose melanomas harbored the *BRAF* V600E mutation [20]. This was an unprecedented result for a melanoma clinical trial and quickly led to the initiation of both phase II and phase III single agent trials [21]. The phase III trial of vemurafenib closed early when the primary progression free survival endpoint was met and the data has been submitted to the FDA for regulatory approval. Although the results from the vemurafenib trial were very impressive, responses were unfortunately short-lived with most patients ultimately failing therapy and becoming resistant (median progression free survival ~7 months) [20]. The development of strategies to manage and overcome acquired BRAF inhibitor resistance is now the major challenge facing the melanoma research community.

The emerging evidence suggests that acquired resistance to vemurafenib is complex and multi-factorial [17, 22-26]. Already, studies have shown that resistance can be mediated via increased receptor tyrosine kinase (RTK) signaling, the acquisition of activating mutations in *NRAS*, novel mutations in MEK1 and up regulation of MAP3K8 (Cot) [22-26]. Although the resistance mechanisms reported thus far are diverse, most are associated with the re-establishment of MAPK signaling and/or an increase in PI3K/AKT/mTOR signaling [22-26]. Clinical trials are currently ongoing to validate the combination of BRAF and MEK inhibitors in *BRAF* V600E mutant melanoma, with trials on the combination of BRAF with AKT inhibitors due to commence in the near future. The end goal of these studies is to define an optimal combination therapy strategy with the aim of extending the time to relapse and improving overall survival.

## USING PROTEOMICS TO UNDERSTAND THE MECHANISMS OF INTRINSIC BRAF INHIBITOR RESISTANCE

Approximately 20% of *BRAF* V600E mutant melanoma patients on the phase I trial of vemurafenib appeared to be intrinsically resistant and did not meet the RECIST criteria for a response [20]. Although uniquely addicted to MAPK signaling, melanomas are also known to require signaling activity in many other pathways, with the PI3K/AKT pathway thought to be particularly important for both melanoma initiation and progression [2, 27, 28]. In a recent study, our lab identified loss of expression of the tumor suppressor phosphatase and tensin homolog (PTEN) as being predictive for an impaired apoptotic response when BRAF was inhibited [29]. Mechanistically it was noted that inhibition of BRAF in PTEN null melanoma cells was associated with an increase in phospho-AKT expression which led in turn to the decreased nuclear accumulation of FOXO3a [29].

As no studies had yet addressed the mechanism by which PTEN expression regulated the apoptotic response following BRAF inhibition, we developed a novel mass spectrometry based technique to simultaneously quantify a large panel of Bcl-2 family proteins. As our approach, we used selected reaction monitoring mass spectrometry (SRM-MS), a method that was originally developed to enable quantification of analytes in complex mixtures. This technology (LC-SRM-MS) has been applied to the detection and quantification of small molecules (MW < 500 Da) for decades, and is used routinely to test for drugs of abuse and performance enhancement and to define pharmacokinetics and pharmacodynamics of therapeutic compounds. It was first applied to endogenous peptides in the early 1990's [30]. However, the use of triple quadrupole mass spectrometers to monitor proteolytic peptides as surrogates for the expression of a protein was first described in 1996 [31]. LC-MRM with the addition of spiked stable isotope labeled peptides enables quantification of the number of moles (or molecules) of the endogenous protein; this method has been termed absolute quantification (AQUA) [32-34]. It is important to note that this technique establishes a minimum value for the amount of the protein as the digestion and peptide recovery are incomplete (although they are extremely consistent).

**Figure 1:**
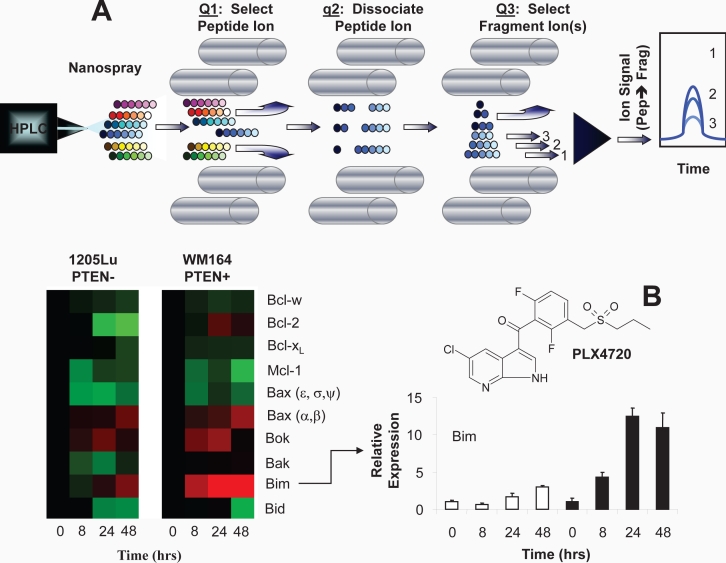
Liquid Chromatography Multiple Reaction Monitoring Mass Spectrometry: Principle and Practice After reversed-phase HPLC separation, peptides are selected by their mass-to-charge ratio and dissociated by collisions with background gas before the fragment ions are mass selected to enable specific detection and quantification of individual peptides in complex mixtures (A). This technique was applied to the measurement of expression of apoptosis-regulating proteins in the Bcl-2 family to determine the mechanism for PTEN null melanoma cells' resistance to the BRAF V600E inhibitor, PLX4720 (inset). Both the heat map and the bar graph indicate that the major difference between the two cell lines was the upregulation of Bim, which caused apoptosis in PTEN positive cells. Regulation of the other Bcl-2 family proteins was similar regardless of PTEN status.

When analyzing multiple fragments from different molecules, the technique is also called multiple reaction monitoring mass spectrometry (MRM-MS). The “reaction” is the conversion of the intact molecule into fragment ion(s) specific for its structure (see Figure 1A); this molecule-fragment pair is also termed a transition. Each “reaction” is optimized by the choice of background gas (typically argon or nitrogen) the pressure in the collision cell, and the collision energy applied. When coupled with reversed-phase liquid chromatography, three characteristics of the molecule are used to isolate its signal for detection and quantification: hydrophobicity (which defines the elution time), the mass-to-charge ratio (m/z) of the intact molecule, and fragment ion mass-to-charge ratio.

This research arena is extremely active and competitive; methods for assessment of panels of protein biomarkers and pipeline development have been described in recent publications [35-39]. Furthermore, LC-MRM shows promise in translation to the assessment of patients. Of equal importance, LC-MRM can be effective in elucidating biological processes; this technology has been used to monitor protein post-translational modifications as well as signaling networks [40-42]. Our study used SDS-PAGE protein fractionation combined with LC-MRM detection and quantification to evaluate the expression of apoptosis-regulating proteins in the Bcl-2 family. The quantification of multiple family members is critical due to the redundancy of their function, and LC-MRM has the capability to measure large numbers of low abundance proteins in a single experiment. Quantification of these proteins revealed a differential up regulation of BIM between the PTEN expressing and PTEN null cells when BRAF was inhibited (14-fold increase vs 4-fold increase, respectively) (Figure 1B) [29].

BIM is a pro-apoptotic BH3-only domain protein that is regulated both transcriptionally and post-transcriptionally by many signaling pathways including BRAF/MEK/ERK, PI3K/AKT, p38 MAPK and JNK/SAPK [43]. It exerts its pro-apoptotic effects by binding to and antagonizing the anti-apoptotic proteins Bcl-2, Bcl-w, Bcl-XL and Mcl-1 [44, 45]. In *BRAF* mutant melanoma cells, inhibition of BRAF using small molecule inhibitors and siRNA knockdown leads to the induction of apoptosis via a mechanism involving the decreased phosphorylation of BAD at Ser-75, an upregulation of BMF and an increase in BIM expression [6, 46, 47]. The identification of BIM as a key PTEN-regulated apoptotic mediator in *BRAF* mutant melanoma cells allowed a novel mechanism of intrinsic drug resistance to be elucidated whereby the paradoxical activation of AKT in PTEN null cells led to a suppression of the nuclear accumulation of FOXO3a and a reduction in BIM mRNA [29]. Of potential clinical relevance it was noted that dual inhibition of both BRAF and PI3K restored nuclear FOXO3a accumulation, upregulated BIM expression at the mRNA and protein levels and enhanced the level of apoptosis [29]. Similar results were also noted in melanoma cells intrinsically resistant to the MEK inhibitor AZD6244, where sensitivity could be restored by the dual inhibition of both MEK and IGF1R, mTORC1/2 or AKT [48]. Further support for the role for AKT signaling in intrinsic BRAF inhibitor resistance came from other studies showing that overexpression of an active form of AKT3 (myristolated AKT3) prevented apoptosis in *BRAF* V600E mutant melanoma cells when BRAF was inhibited [49].

The use of LC-MRM allowed the level of BIM expression to be identified as a PTEN-dependent determinant of BRAF inhibitor mediated apoptosis. If it were not for the exquisite sensitivity of the LC-MRM approach and the ability to accurately quantify peptide levels, the PTEN dependency of BIM expression in this process may not have been realized. The utility and value of LC-MRM comes primarily from the fact that experiments can be designed to evaluate the full complexity and detail of biological processes in different human disease states.

## CURRENT VALUE AND PROJECTED FUTURE POTENTIAL OF LC-MRM PROTEIN QUANTIFICATION

Quantitative mass spectrometry, particularly LC-MRM, is emerging as an alternative to antibody-based methods for the detection and quantification of proteins (such as Western Blotting). The development of peptide-based assays for protein expression, modification, and even mutations can be implemented very rapidly [50]. The selection of the target peptide sequence from either existing tandem mass spectrometry data or LC-MRM screening of peptide candidates, synthesis of stable isotope-labeled standards, and analysis to examine the match between the elution time and fragmentation patterns of the endogenous and standard peptide can be completed within a few days. Current mass spectrometers can analyze hundreds to thousands of transitions (peptide and fragment pairs) in each experiment enabling quantification of numerous proteins. While one peptide could be used as a surrogate, typically three or more transitions from three or more peptides are recommended for quantifying protein expression. Unlike antibody-based methods that are completely reliant on the reagents and the epitopes that they recognize, protein measurements with LC-MRM have a great deal more flexibility [51]. If an assay has interference, additional steps can be taken to change the protein purification, select another peptide, or even just select a different fragment ion from the same peptide. Through its versatility and capacity for multiplexing, LC-MRM platforms should soon emerge as competitors for array technologies. Just as peptide sequencing with liquid chromatography-tandem mass spectrometry (LC-MS/MS) has revolutionized biology, the ability of LC-MRM to detect and quantify low abundance targets determined a priori represents the next potential impact that proteomics can have on the study of human disease. As we have demonstrated, these methods have particular utility in unraveling how the genetic makeup of cancer cells can dictate drug response. As cancer therapy becomes ever more personalized, and the LC-MRM technology becomes more sophisticated, a future can be envisioned in which the key determinants of drug response can be determined in individual patient samples. It is hoped that these new approaches could allow therapies to be specifically tailored to individual patients so that efficacy can be maximized and off-target effects minimized.
